# Stigmasterol, the Active Ingredient in Huanglian Decoction, Inhibits the MAOA-NF-κB-MLCK Pathway to Improve the Inflammation in Rats With Reflux Esophagitis

**DOI:** 10.2174/0118715303432369260203080405

**Published:** 2026-04-08

**Authors:** Huan Wang, Lin Li, Jian Han, Yan Li, Jun Yang, Wangzhong Qiu, Liuhua Yao

**Affiliations:** 1 Department of Spleen and Stomach Diseases, Yueyang Hospital of Traditional Chinese Medicine, Yueyang, China;; 2 Department of Laboratory, Yueyang Hospital of Traditional Chinese Medicine, Yueyang, China

**Keywords:** Reflux Esophagitis, *Huanglian* decoction, MAOA, NF-κB-MLCK pathway

## Abstract

**Objective:**

Traditional Chinese medicine (TCM) shows significant potential in treating reflux oesophagitis (RE). *Huanglian* Decoction (HD) is a well-known classic TCM. This study aimed to investigate the active constituents, specifically Stigmasterol and the underlying mechanisms through which HD exerts its therapeutic effects on RE.

**Methods:**

We employed network pharmacology, metabolomics, and molecular docking to analyze the active ingredients and interaction targets of HD and RE. An *in vitro* model of RE was created by incubating Het-1A cells with acidified BEBM medium. The effects of HD and Stigmasterol were evaluated by measuring cell viability, levels of inflammatory factors, and expression of tight junction proteins. Finally, the validity of the *in vitro* findings was confirmed by assessing the esophageal damage and inflammation in RE rats.

**Results:**

Network pharmacology has identified monoamine oxidase A (MAOA) as a core target of HD in the treatment of RE. Metabolomic analysis revealed Stigmasterol to be the active component of HD. Functional studies demonstrated that HD and Stigmasterol enhanced cell viability in acid-exposed conditions, mitigated inflammation, and increased the expression of occludin, claudin-1, and ZO-1, while simultaneously reducing levels of p-p65, MLCK, and MAOA. Molecular docking revealed that stigmasterol binds to MAOA. Notably, the protective effect of Stigmasterol on Het-1A was negated by the overexpression of MAOA. Furthermore, *in vivo* studies demonstrated that HD and Stigmasterol improved acute RE in rats by inhibiting the MAOA/NF-κB-MLCK axis.

**Discussion:**

This study elucidates the role and mechanism of HD in RE. However, further clinical trials are needed to assess its applicability in clinical practice.

**Conclusion:**

Stigmasterol, the active ingredient in HD, inhibits the NF-κB-MLCK pathway via MAOA, thereby reducing RE-related cellular inflammation. This research offers novel insights for RE treatment.

## 1 INTRODUCTION

Reflux oesophagitis (RE) is a chronic inflammatory disease of the oesophageal mucosa, resulting from gastroesophageal reflux disease (GERD) [1]. The global prevalence of RE is on the rise, leading to a notable decrease in the quality of life for those affected and placing a substantial financial strain on society [2]. Proton pump inhibitors (PPIs) are the preferred pharmacological agents for managing and preventing gastroesophageal reflux. However, approximately 10% to 40% of patients with gastroesophageal reflux do not respond adequately to PPIs, indicating the presence of refractory gastroesophageal reflux [3]. Consequently, it is essential to explore new therapeutic options for treating RE.

Traditional Chinese medicine (TCM) prescriptions, which prioritize a holistic approach and adhere to the principles of syndrome differentiation and treatment, have demonstrated considerable promise and advantages in addressing RE [4, 5]. *Huanglian* Decoction (HD), a classic TCM decoction, is composed of various herbal components, including *Coptis chinensis Franch.*, *Pinellia ternata (Thunb.) Makino*, and *Glycyrrhiza uralensis Fisc* [6]. Previous studies have shown that *Coptis chinensis Franch,* along with other TCM combinations, could be a viable alternative therapy for esophageal cancer [7]. Still, the detailed role of Huanglian Decoction in addressing Reflux Esophagitis requires further exploration.

Research suggests that dysfunction of the esophageal epithelial barrier may contribute to the development of RE [8]. Additionally, an upregulation of pro-inflammatory factors is a key pathological feature of RE [9, 10]. Considering that HD plays a pharmacological role in maintaining intestinal barrier integrity and is implicated in ulcerative colitis [11], we hypothesize that HD may inhibit RE by exerting anti-inflammatory effects and promoting the repair of epithelial barrier lesions.

Studies have shown that MAOA regulates the expression of nuclear factor kappa B subunit 1 (NF-κB) in neurodevelopmental toxicity [12]. Furthermore, the NF-κB pathway mediates the activation of the myosin light chain kinase (MLCK) signaling pathway in cases of intestinal epithelial barrier damage [13]. Significantly, the NF-κB pathway is associated with esophageal inflammation induced by N-methyl-N'-nitro-N-nitrosoguanidine [14]. A growing body of research indicates that HD and its herbal mixtures can alleviate different inflammatory conditions by influencing the NF-κB signaling pathway. For example, HD has been shown to improve nephritis by inhibiting the NF-κB/NLR family pyrin domain-containing 3 (NLRP3) signaling pathway [15]. However, further research is needed to determine whether HD can alleviate the progression of RE by inhibiting the NF-κB/MLCK signaling pathway through the action of MAOA.

Foods or supplements enriched with phytosterols hold promise as potential chemopreventive agents for esophageal cancer [16]. Among these compounds, stigmasterol is a notable phytosterol [17]. Our study identified stigmasterol as a crucial pharmacological component within the HD-RE regulatory network, with MAOA recognized as one of the primary target genes associated with HD in the treatment of reflux esophagitis (RE). To further explore the cellular mechanisms by which HD exerts its effects on RE, we developed an acid model using Het-1A cells based on previous research [9]. Upon treatment with HD, we found that stigmasterol effectively inhibits the activation of the NF-κB-MLCK pathway by targeting the MAOA protein. This inhibition leads to anti-inflammatory effects and the repair of tight junctions, ultimately ameliorating the damage associated with RE. In summary, our findings suggest that stigmasterol, an active component of HD, enhances the condition of RE by inhibiting the MAOA/NF-κB/MLCK axis. This research offers new theoretical insights for the treatment and management of GERD.

## 2 MATERIALS AND METHODS

### 2.1 Target Acquisition and Network Construction

The TCM Systems Pharmacology Database and Analysis Platform (TCMSP) (https://tcmspw.com/tcmsp.php) was employed to investigate the ingredients and targets of HD (*Coptis chinensis Franch., Pinellia ternata (Thunb.) Makino, cinnamomi ramulus, Codonopsis*, *Dried Ginger*, *Glycyrrhiza uralensis Fisc, Ziziphus jujuba Mill.*). A keyword search for “Gastroesophageal reflux” was conducted across multiple databases, including GeneCards database (https://www.genecards.org/), the National Center of Biotechnology Information (NCBI) gene database (https://www.ncbi.nlm.nih.gov/), and the DisGeNET database (https://www.disgenet.org/). After compiling the identified genes, duplicates were removed, resulting in the final list of target-related genes. Venny 2.1 software was utilized to identify shared targets between HD and RE. Subsequently, a protein-protein interaction (PPI) network was established using Cytoscape, followed by topology and MCODE cluster analysis.

### 2.2 High-Performance Liquid Chromatography-Tandem Mass Spectroscopy (HPLC-MS) Analysis

Methanol and 2-Chloro-L-phenylalanine (103616-89-3, Hengbai, China) were added to the HD sample and swirled and mixed for 35 seconds. The mixture was then subjected to ultrasound treatment, allowed to stand, and subsequently centrifuged. The components of the collected supernatant were analyzed using HPLC-MS. The samples were analyzed using a C18 column (150 × 4.6 mm, 5 μm, Thermo, USA). Formic acid (A) and acetonitrile (B) served as the mobile phases. The gradient elution strategy was as follows: before injection, the mobile phase consisted of 3% B for 0-1 mins, 20% B for 1-2 min, 45% B for 2-4 min, 70% B for 4-10 min, 20% B for 10-15 min, 5% B for 15-15.1 min. The column was then equilibrated for 5 min. Chromatographic separations were performed using a 10 μL injection volume and a flow rate of 0.6 mL/min. The HD sample was analyzed in positive electrospray ionization (ESI) mode. The data were obtained by monitoring the selected ions in multi-reaction monitoring (MRM) mode.

### 2.3 Molecular Docking Validation

The three-dimensional structure diagram of Stigmasterol was obtained from the PubChem database (https://pubchem.ncbi.nlm.nih.gov/). A structural model of stigmasterol was produced using Chem3D Pro and subsequently saved in a Mol2 file format. The 3D structure of MAOA was retrieved from the RCSB Protein Data Bank (PDB) (https://www.rcsb.org/). Molecular docking was performed utilizing VINA version 1.1.2.

### 2.4 Cell Origin and Construction of RE *in vitro* Models

The human esophageal epithelial cell line Het-1A (AW-CNH217) was procured from Changsha Abiowell Biotechnology Co., Ltd. (China). The cells were cultured in a specialized medium tailored for Het-1A cells (AW-MNH217, Abiowell). An acid model of RE was created by acidifying the BEBM medium with 3% hydrochloric acid and incubating Het-1A monolayer cells in the acidified medium for 30 min [18].

### 2.5 Cell Grouping and Treatment

To determine the optimal incubation concentration and duration for HD treatment, different concentrations of HD were added to acidified culture medium-induced Het-1A cells [19]. The experimental groups were defined as follows: Normal group: Cells were grown under standard conditions. Model group: A model of RE was constructed by incubating Het-1A monolayer cells in acidified BEBM for 30 min. HD-L/M/H groups: Acid-treated Het-1A cells were incubated with HD-L (5 mL, 0.195 mg/mL), HD-M (5 mL, 0.39 mg/mL), and HD-H (5 mL, 0.78 mg/mL) for 24 h each.

A concentration of 1, 5, or 10 μM of Stigmasterol was applied to Het-1A to determine the optimal concentration [20]. Normal group and Model group as described above, Stigmasterol group: Het-1A acid model were treated with Stigmasterol-H (10 μM) for 24 h.

To further explore the relationship and functionality of Stigmasterol and Monoamine oxidase A (MAOA) in RE, we performed the grouping process described below. Stigmasterol+oe-NC group: Stigmasterol-L (10 μM) was added to the acid-treated Het-1A cells, and at the same time, oe-NC was transfected for 24 h. Stigmasterol+oe-MAOA group: Stigmasterol-L (10 μM) was added to the acid-treated Het-1A cells, and at the same time, oe-MAOA was transfected for 24 h.

To further explore the relationship and functionality of NF-κB and MAOA in RE, we performed the grouping process described below. oe-NC group: Acid-treated Het-1A cells were transfected with oe-NC. oe-MAOA group: Acid-treated Het-1A cells were transfected with oe-MAOA. oe-MAOA+QNZ group: A combination of oe-MAOA transfection and treatment with the NF-κB inhibitor (QNZ, 10 μM) was applied to the Het-1A acid model for 24 h.

Overexpression vectors for MAOA (oe-MAOA) and a control (oe-NC) were synthesized by HonorGene (China). The Lipofectamine 2000 transfection kit (Invitrogen, USA) was used to transfect oe-MAOA and oe-NC into Het-1A cells, respectively. The cells were collected 24 h after transfection.

### 2.6 Cell Counting Kit-8 (CCK-8)

Cells were seeded at a density of 3×10^3^ cells per well in 96-well plates. CCK-8 (AWC0114s, Abiowell) was pipetted into the wells at 10 μL/well. The plates were incubated at 37°C in a 5% CO2 atmosphere for 4 h. After incubation, the optical density (OD) values at 450 nm were measured using a microplate reader (HEALES, China).

### 2.7 Quantitative Reverse Transcription PCR (qRT-PCR)

RNA was extracted using Thermo's RNA extraction kit (15596262, USA). A Reverse Transcription Reagent Kit was used to perform reverse transcription on the extracted RNA (15596026, CWBio, China). The cDNA was amplified with an UltraSYBR Mixture (CW2601, CWBio). Data analysis was carried out using the 2^-ΔΔCt^ method and normalized to β-actin. The sequence of primers is shown in Table **1**.

### 2.8 Enzyme-Linked Immunosorbent Assay (ELISA)

Cell samples were centrifuged at 1000g for 15 min at 2-8°C. Approximately 100 mg of esophageal mucosal tissue was minced into small pieces, homogenized, and then centrifuged at 5000g for 5 min at 2-8°C. The supernatants from both the cell and tissue homogenates were collected for further analysis. The levels of MAOA(human: CSB-E10144h, CUSABIO; rat: CSB-E15076r, CUSABIO), tumor necrosis factor-alpha (TNF-α, human: CSB-E04740h, CUSABIO; rat: KE20018, Proteintech), interleukin-1β (IL-1β, human: CSB-E08053h, CUSABIO; rat: KE20021, Proteintech), interleukin-6 (IL-6, human: CSB-E04638h, CUSABIO; rat: CSB-E04640r; CUSABIO) and interleukin-8 (IL-8, human: CSB-E04641h, CUSABIO; rat: ml037351v, mlbio) in the cell supernatant and esophageal mucosa were measured using specified kits. With a microplate reader, OD (450 nm) was measured.

### 2.9 Western Blot

Total protein was extracted utilizing RIPA lysis buffer (AWB0136, Abiowell). The concentration of the sample protein was quantified using a BCA protein quantitation kit (P0010S, Beyotime, China). Separated proteins were electrophoresed on SDS-polyacrylamide gels electrophoresis (SDS-PAGE) and then transferred to nitrocellulose membranes. The membrane was blocked with 5% skim milk powder (AWB0004, Abiowell).

The nitrocellulose membranes were incubated overnight at 4°C with primary antibodies, including occludin (27260-1-Ap, Proteintech, USA), claudin-1 (28674-1-Ap, Proteintech), ZO-1 (21773-1-Ap, Proteintech), MAOA (10539-1-Ap, Proteintech), p-p65 (AWA47471, Abiowell), p65 (AWA00848, Abiowell), MLCK (ab314185, Abcam, UK), and β-actin (AWA80002, Abiowell).

Then, the nitrocellulose membranes were incubated with the second antibody, Goat anti-Rabbit IgG H+L (HRP) (AWS0002, Abiowell) or Goat anti-Mouse IgG H+L (HRP) (AWS0001, Abiowell). After incubation, SuperECL Plus Luminous Fluid (AWB0005, Abiowell) was administered to the membrane and incubated for 1 min. The membrane was imaged using a gel imaging system (ChemiScope6100, Shanghai Qingxiang, China).

### 2.10 The RE Rats Model and Drug Management

Male Sprague-Dawley rats (7 weeks old, 180±20 g, SJA, China) were housed in standard rat cages within the facility, with free access to food and water provided. A 12 h light/dark cycle is to be maintained, and the temperature is to be controlled at 21-25°C, with a humidity range of 35-60%.

After a 7-day adaptation period, these 8-week-old rats were randomly divided into 5 experimental groups (n = 5): a Normal group, a RE group, an HD group, a Stigmasterol group, and a Ranitidine group (positive control). RE rats were anesthetized by intraperitoneal injection of 30 mg/kg of pentobarbital sodium. A 2-centimeter incision was made on the abdomen of the rat to expose the stomach. The connections between the stomach, the Forestomach, and the pylorus were all bound with 3-0 silk thread. It is necessary to ensure that the vagus nerve remains intact [21].

The rats were fasted for 18 h before the operation. The rats in the normal group did not receive oral administration, while the rats in the RE control group were given oral saline. Rats in the drug group were orally administered HD [22] and Stigmasterol [23] at doses of 200 mg/kg body weight and 40 mg/kg body weight, respectively. In contrast, rats in the positive control group were orally administered ranitidine at a dose of 40 mg/kg body weight [21]. After 2 h of drug treatment, these rats were used to construct the RE model.

All the experimental rats were euthanized 4.5 h after the surgery by intraperitoneal injection of 150 mg/kg of pentobarbital sodium. The esophageal and gastric tissues were placed on paper, and the esophageal mucus was washed with 0.9% saline. Then, digital photos were taken. Esophageal injury was analyzed using ImageJ software. The calculation of the esophageal lesion index is as follows: Lesion index (%) = (Esophageal injury area (mm^2^)/Total esophageal area (mm^2^))×100. The esophageal tissue was stored at -80°C until analysis.

### 2.11 Hematoxylin-Eosin (H&E) Staining

The paraffin sections of esophageal tissue were placed in xylene for 20 min (three times), then dehydrated successively in 100%, 100%, 95%, 85%, and 75% ethanol. The sections were stained with hematoxylin (AWI0001a, Abiowell) for 10 min, followed by staining with eosin (AWI0029a, Abiowell) for 5 min. Then, they were dehydrated using a gradient of ethanol (95-100%). Finally, they were sealed with neutral resin and observed under a light microscope for esophageal damage.

### 2.12 Ethics Approval and Consent to Participate

This study strictly adhered to the ARRIVE guidelines and the “Guidelines for the Care and Use of Laboratory Animals”. The research design has minimized the number of animals used (n = 5 per group), and the sample size has been determined based on previous studies [24]. All the protocols for the research project have been approved by Hunan University of Chinese Medicine Experimental Animal Ethics Committee (Ethics number:20231109).

### 2.13 Data Statistics and Analysis

GraphPad Prism 8 software was used for statistical analysis of the data. A *t*-test was utilized to compare the two groups. A one-way or two-way analysis of variance was used to compare multiple groups, and the Tukey post hoc test was employed to make pairwise comparisons. *P* < 0.05 was considered statistically significant.

## 3 RESULT

### 3.1 Analysis of Interaction Targets Between HD and RE

**Fig. (1):**
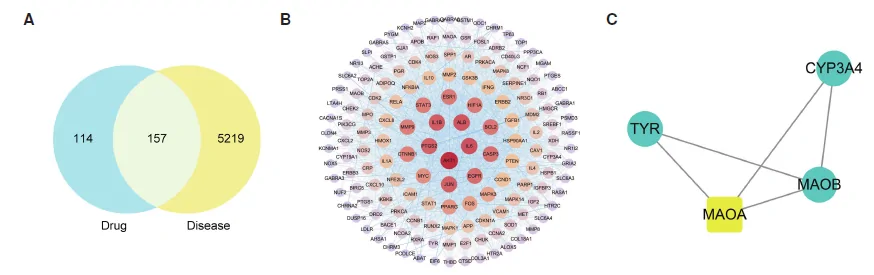
Analysis of interaction targets between HD and RE. (**A**) Venn diagram showing common targets of drugs and diseases. (**B**) The PPI network shows target gene interaction. (**C**) MCODE cluster analysis.

To explore the target of HD in RE, we utilized network pharmacology to analyze the common targets of HD and RE. Through screening, we identified 271 high-density (HD) drug targets and 5376 receptor-expressed (RE) disease-related targets. As shown in Fig. (**1A**), 157 of these targets were found to be common between HD and RE, making them potential targets of HD for RE. These findings led us to construct a PPI network diagram of HD-RE-target (Fig. **1B**). Then, we found that MAOA was one of the core target genes of HD and RE (Fig. **1C**). These results suggest that MAOA is a promising target for intervention in RE.

### 3.2 HD Relieves Inflammation in the Context of RE and Inhibits the Expression of MAOA

**Fig. (2):**
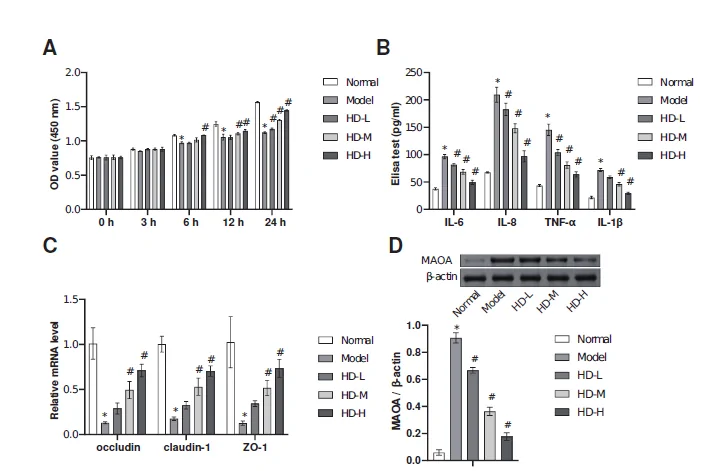
HD relieves inflammation in the context of RE and inhibits the expression of MAOA. (**A**). Cell viability was evaluated using the CCK-8 assay. (**B**) ELISA performed the expression of IL-6, IL-8, TNF-α, and IL-1β. (**C**) The occludin, claudin-1, and ZO-1 mRNA levels were assessed with qRT-PCR. (**D**) The MAOA protein levels were detected using a Western blot. n=3, * *P* < 0.05 *VS* Model group. # *P* < 0.05 *VS* Model group.

To validate the effects of HD in RE at the cellular level, we used an acidic culture medium to incubate Het-1A and constructed a cellular acid model of RE. Then, these cells were treated with different concentrations of HD. We performed the CCK-8 assay to measure cell viability at 0, 3, 6, 12, and 24 h. The results exhibited that after 24 h of treatment with HD-L, HD-M, and HD-H, the cell viability inhibited by an acidic culture medium was substantially restored (Fig. **2A**). The ELISA assay revealed that after 24 h of treatment with HD-L, HD-M, and HD-H, the levels of IL-6, IL-8, TNF-α, and IL-1β in the cell supernatant were significantly decreased compared to the Model group (Fig. **2B**). The results of qRT-PCR showed that mRNA levels of occludin, claudin-1, and ZO-1 in HD-M and HD-H cells increased significantly after 24 h of treatment compared with the Model group (Fig. **2C**). In addition, after treating cells with HD, the protein levels of MAOA were significantly reduced in cells compared with the Model group (Fig. **2D**). These results demonstrated that HD alleviated the damage to Het-1A cells in the context of RE and inhibited the expression of MAOA.

### 3.3 Metabolomic and Molecular Docking Analysis of the Binding Between HD Components and Target MAOA

**Fig. (3):**
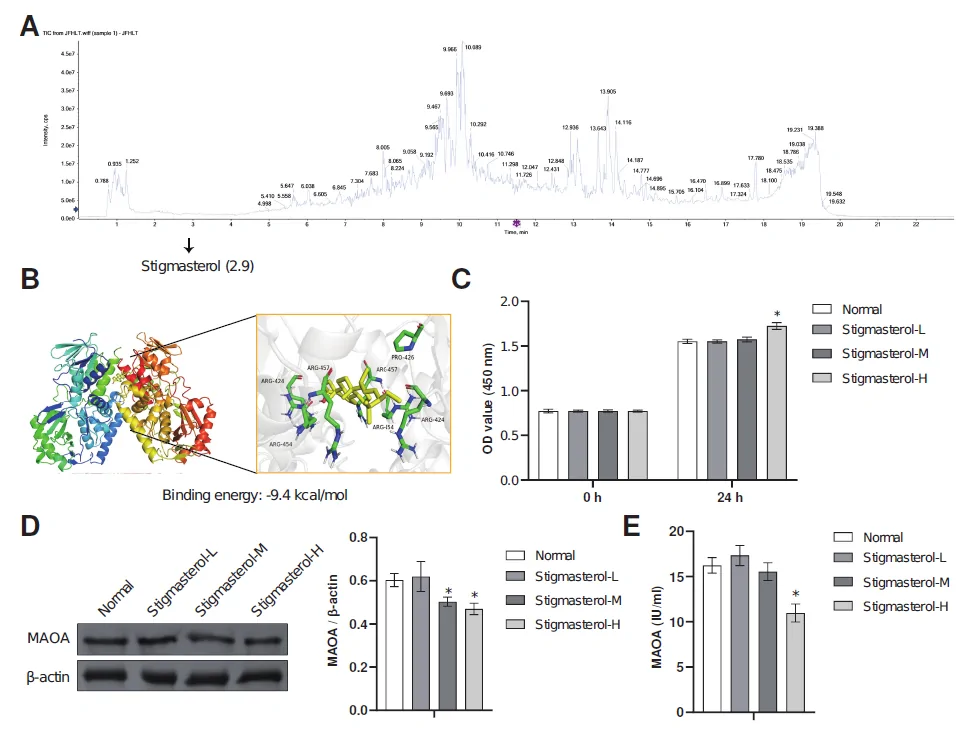
Metabolomics and molecular docking analysis of HD drug component binding with the protein of the target gene. (**A**) HPLC-MS was utilized to analyze the pharmaceutical HD drug components. (**B**) Molecular docking technology was used to analyze the binding energy of the active component stigmasterol with the core target protein MAOA. (**C**) CCK-8 was used to analyze the cell viability. (**D**) The MAOA protein expression in cells was quantified by Western blot. (**E**) ELISA was used to detect the activity of MAOA. n=3, * *P* < 0.05 *VS* Normal group.

To investigate the components of HD acting on RE, HPLC-MS analysis was conducted on the HD samples. The results showed that the Expected Retention Time (RT) of Stigmasterol was 2.9 minutes, and the actual detected Retention Time was also 2.9 minutes, showing a high degree of consistency. This strongly confirmed the presence of Stigmasterol in the HD samples. At the same time, the peak area (Area) reached 78730, and the peak height (Height) was 6615, indicating that the content of this compound in the samples was relatively considerable and the detection signal was strong (Fig. **3A** and Supplementary Tables **1**). However, the role of Stigmasterol in RE is unknown. In addition, molecular docking analysis confirmed the binding relationship between Stigmasterol and MAOA target proteins (Fig. **3B**). To further understand the protective role of Stigmasterol in RE and the function of MAOA, we determined the optimal treatment concentration to be stigmasterol-H (10 μM) (Fig. **3C**). Also, after treatment with stigmasterol-M (5 μM) and stigmasterol-H (10 μM), the protein expression of MAOA in Het-1A cells was significantly reduced (Fig. **3D**). The ELISA results showed that Stigmasterol-H (10 μM) inhibited MAOA activity (Fig. **3E**). These results suggest that Stigmasterol, a practical component of HD, may improve RE by targeting MAOA.

### 3.4 The Effective Component Stigmasterol of HD Suppressed Inflammation in the Context of RE by Targeting the MAOA

**Fig. (4):**
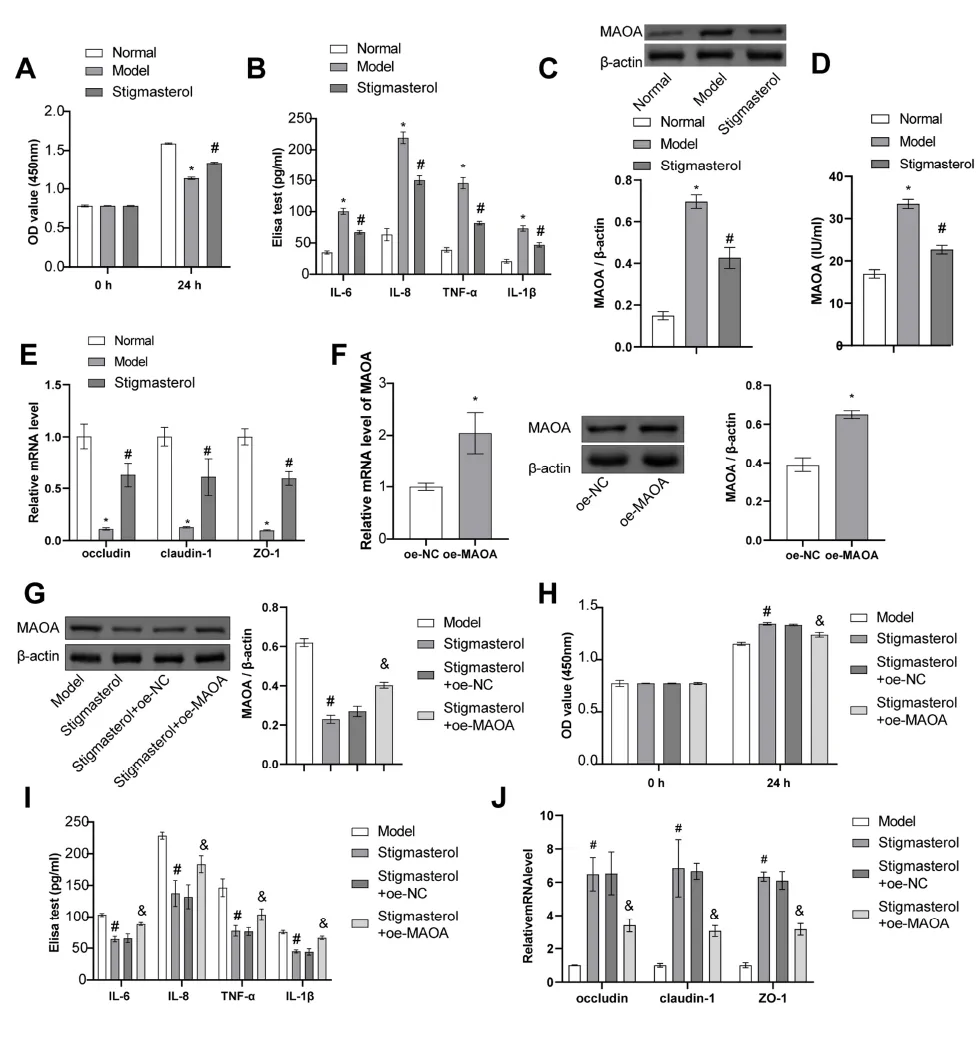
The effective component stigmasterol of HD could suppress inflammation in the context of RE by targeting the MAOA. (**A**) We utilized CCK-8 analysis to determine cell viability. (**B**) We used ELISA to evaluate the levels of IL-6, IL-8, TNF-α, and IL-1β. (**C**) We utilized a Western blot to quantify MAOA expression. (**D**) ELISA was used to detect the activity of MAOA. (**E**) QRT-PCR quantified the mRNA level of occludin, claudin-1, and ZO-1. (**F**) QRT-PCR and Western blot were performed to examine the expression of MAOA. (**G)** Western blot was utilized to examine the expression of MAOA. (**H**) CCK-8 was performed to evaluate the cell viability. (**I**). Secretion levels of IL-6, IL-8, TNF-α, and IL-1β were analyzed using ELISA. (**J**). QRT-PCR analyzed the occludin, claudin-1, and ZO-1 mRNA levels. n=3, * *P* < 0.05 compared with Normal or oe-NC group. # *P* < 0.05 *VS* Model group. & *P* < 0.05 *VS* stigmasterol+oe-NC group.

To investigate the function of Stigmasterol in the improvement of RE, we added 10 μM stigmasterol to the Het-1A acid model. The results of CCK-8 showed that after treatment with Stigmasterol, the cell vitality inhibited by acid treatment was visibly improved compared with the Model group (Fig. **4A**). ELISA results showed that after treatment with Stigmasterol, the levels of IL-6, IL-8, TNF-α, and IL-1β in cell supernatant were significantly reduced compared with the Model group (Fig. **4B**). Western blot results showed that after treatment with Stigmasterol, the expression of MAOA was substantially decreased compared with the Model group (Fig. **4C**). The ELISA results further confirmed that Stigmasterol inhibited the expression of MAOA (Fig. **4D**). QRT-PCR results showed that after treatment with Stigmasterol, the mRNA levels of occludin, claudin-1, and ZO-1 were markedly increased compared with the Model group (Fig. **4E**). These results suggest that Stigmasterol relieved the damage to Het-1A cells in the RE context.

To further explore the role of Stigmasterol in enhancing RE through MAOA, we transfected oe-MAOA into Het-1A cells and validated the transfection efficiency (Fig. **4F**). Next, we treated the Het-1A acid model with stigmasterol while concurrently transfecting oe-MAOA. The results from Western blot analysis revealed a significant reduction in MAOA levels following stigmasterol treatment compared to the Model group. After the transfection of oe-MAOA, we observed a marked increase in MAOA expression compared to the stigmasterol + oe-NC group (Fig. **4G**). CCK-8 analysis revealed a significant decrease in cell viability after MAOA overexpression compared to the stigmasterol + oe-NC group (Fig. **4H**).

Additionally, ELISA results demonstrated that the overexpression of MAOA significantly elevated the secretion levels of IL-6, IL-8, TNF-α, and IL-1β in the cell supernatant relative to the stigmasterol+oe-NC group (Fig. **4I**). Moreover, A qRT-PCR analysis showed that, compared with the stigmasterol+oe-NC group, overexpression of MAOA significantly reduced the mRNA levels of occludin, claudin-1, and ZO-1 (Fig. **4J**). These results demonstrated that Stigmasterol suppressed inflammation in the RE context by targeting MAOA.

### 3.5 The Effective Component Stigmasterol of HD Suppressed Inflammation in the Context of RE by Targeting the MAOA/NF-κB-MLCK Axis

**Fig. (5):**
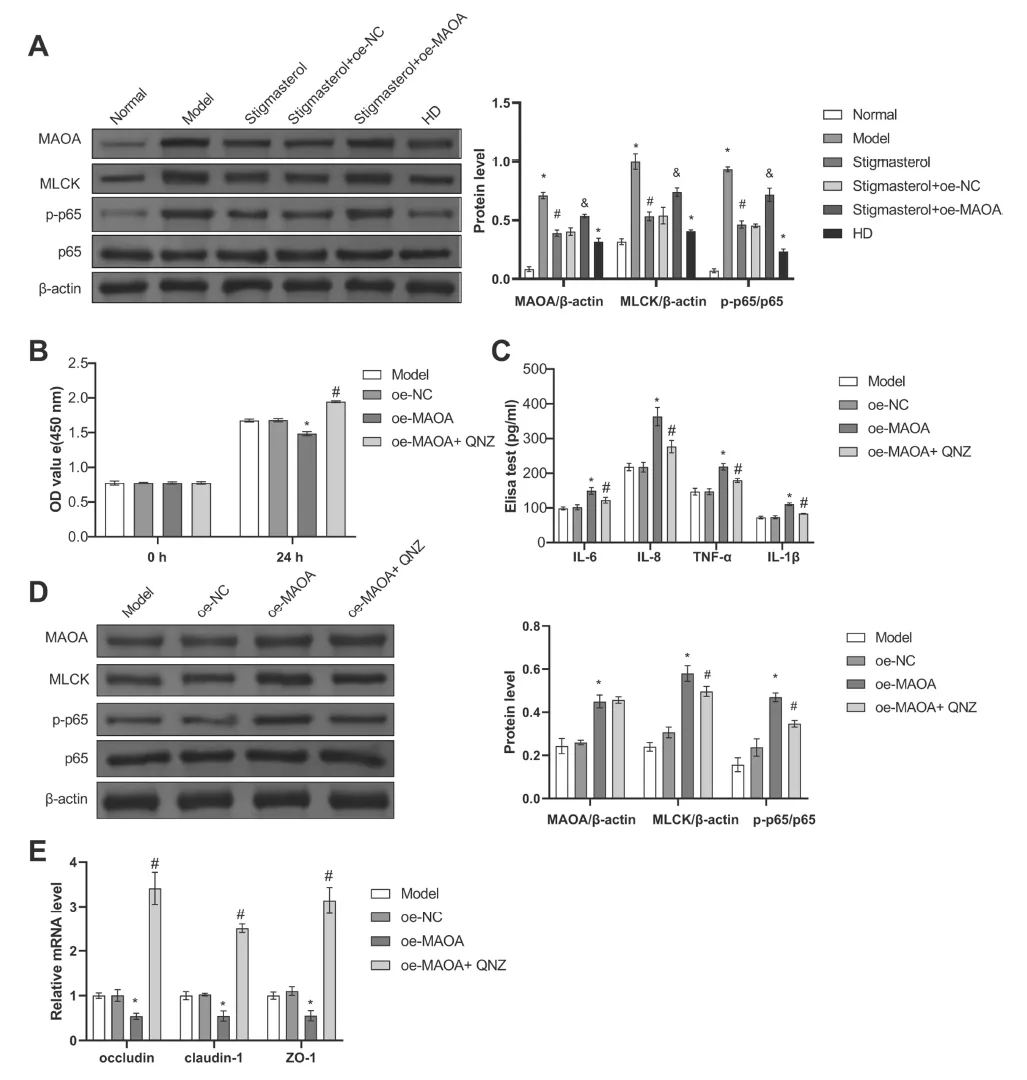
The effective component stigmasterol of HD could suppress inflammation in the context of RE by targeting the MAOA/NF-κB-MLCK axis. (**A**) Western blot was employed to examine the protein expression of MAOA, p-p65/p65, and MLCK. (**B**) CCK-8 detection of cell viability. (**C**) ELISA was performed to examine the levels of IL-6, IL-8, TNF-α, and IL-1β. (**D**) Protein of MAOA, p-p65/p65, and MLCK expression were analyzed using Western blot. (**E**) qRT-PCR was used to detect mRNA levels of occludin, claudin-1, and ZO-1. n=3, * *P* < 0.05 *VS* Normal or oe-NC group. # *P* < 0.05 *VS* Model or the oe-MAOA group. & *P* < 0.05 *VS* stigmasterol+oe-NC group.

It has been reported that the NF-κB-MLCK pathway is associated with intestinal epithelial injury [25]. Therefore, we investigated the role of the NF-κB-MLCK pathway in the amelioration of damage in the context of RE by targeting the MAOA protein with Stigmasterol. Western blot detection results show that the protein levels of MAOA, p-p65/p65, and MLCK were significantly increased in the Model group cells compared with the Normal group. After treatment with Stigmasterol or HD, the protein levels of MAOA, p-p65/p65, and MLCK were significantly decreased compared with the Model group. However, the overexpression of MAOA significantly reversed the effect of Stigmasterol on the expression of several NF-κB-MLCK pathway-related proteins compared with the stigmasterol+oe-NC group (Fig. **5A**). These results suggest that HD and Stigmasterol may inhibit RE by targeting the MAOA/NF-κB-MLCK pathway.

Oe-MAOA was transfected into the acid model cell, or the NF-κB inhibitor QNZ was added at the same time. Functional analysis revealed that overexpression of MAOA further inhibited cell viability and increased the secretion levels of IL-6, IL-8, TNF-α, and IL-1β in the cell supernatant compared with the oe-NC group. However, the addition of QNZ reversed the influence of MAOA overexpression on cell viability and cytokine levels (Figs. **5B** and **5C**). Compared with the oe-NC group, the expression of MAOA, p-p65/p65, and MLCK was further enhanced after MAOA overexpression (Fig. **5D**). In addition, compared with the oe-NC group, the mRNA levels of occludin, claudin-1, and ZO-1 were further inhibited after MAOA overexpression (Fig. **5E**). Compared with the oe-MAOA group, the expression of p-p65/p65 and MLCK in the oe-MAOA+QNZ group significantly decreased, and the expression of occludin, claudin-1, and ZO-1 significantly increased (Figs. **5D** and **5E**). These results demonstrated that HD inhibited RE by targeting the MAOA/NF-κB-MLCK pathway.

### 3.6 HD and its Active Ingredient, Stigmasterol, Improved the Injury of Acute RE in Rats

**Fig. (6):**
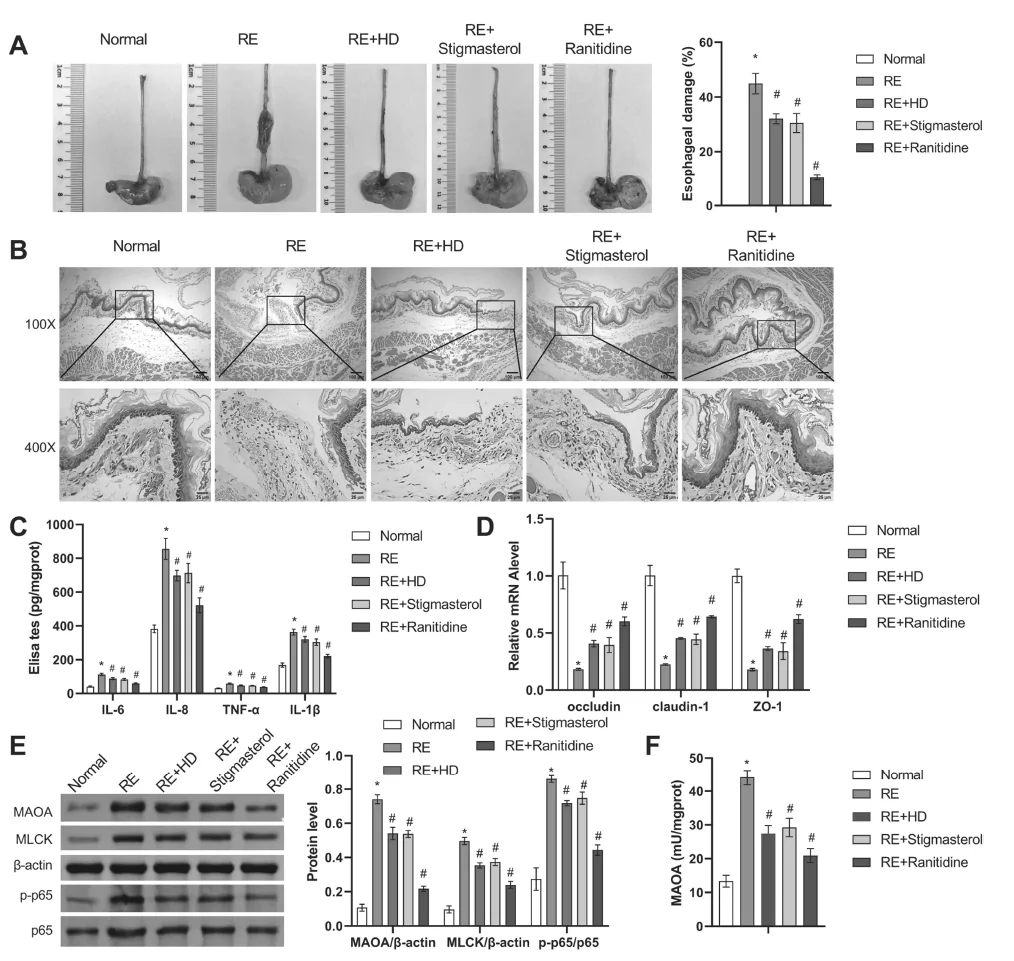
HD and its active ingredient, Stigmasterol, improve the injury of acute reflux esophagitis in rats. (**A**) Esophageal damage index. (**B**) H&E staining was used to observe the pathological damage of esophageal tissues. The contents of TNF-α, IL-1β, IL-6, and IL-8 in esophageal mucosa were detected by (**C**) ELISA. (**D**). The expressions of occludin, claudin-1, and ZO-1 in esophageal tissues were detected by qRT-PCR. (**E**) The expression levels of proteins MAOA, p-p65/p65, MLCK, and MLC in esophageal tissues were detected by Western Blot. (**F)** ELISA was used to detect the activity of MAOA. n=5, * *P* < 0.05 *VS* Normal group, # *P* < 0.05 *VS* RE group.

Finally, we explored the effect of HD and Stigmasterol in the treatment of RE in rats. As shown in Fig. (**6A**). Compared with the Normal group, the RE group showed severe congestion and ulcers, and the rate of esophageal injury was very high. After oral administration of HD, Stigmasterol, and Ranitidine, the rate of esophageal injury was significantly reduced. Observation by H&E staining showed that the esophageal mucosal structure in the Normal group was standard. In the RE group, the esophageal mucosa showed a large amount of diffuse inflammatory cell infiltration. Compared with the RE group, the inflammatory cell infiltration in the esophageal mucosa of RE rats orally administered HD, Stigmasterol, and Ranitidine was all alleviated (Fig. **6B**). The ELISA test results showed that compared with the Normal group, the contents of IL-6, IL-8, TNF-α, and IL-1β in the RE group were significantly increased (Fig. **6C**). The results of qRT-PCR detection showed that compared with the Normal group, the mRNA levels of occludin, claudin-1, and ZO-1 in the esophageal mucosal tissues in the RE group were significantly decreased (Fig. **6D**).

Furthermore, Western Blot detection results showed that, compared with the Normal group, protein levels of MAOA, p-P65, and MLCK were significantly increased in the RE group (Fig. **6E**). Subsequently, the ELISA test results further confirmed the changes in MAOA expression (Fig. **6F**). However, after oral administration of HD, Stigmasterol, and Ranitidine, these changes were partially reversed (Figs. **6C**-**6F**). These data indicated that HD and its active ingredient, Stigmasterol, improved esophageal mucosal injury caused by RE through the MAOA-NF-κB-MLCK pathway.

## 4 DISCUSSION

RE frequently occurs due to the backflow of gastric acid and other stomach materials. Its development is strongly linked to an immune-inflammatory reaction and the malfunction of the esophageal epithelial barrier [10, 26]. In this study, we identified the primary target of the HD-RE regulatory network as MAOA, with Stigmasterol serving as the active component. Through both *in vitro* and *in vivo* studies, we demonstrated that HD and its key active component, Stigmasterol, inhibited the activation of the NF-κB/MLCK pathway by targeting MAOA. This resulted in anti-inflammatory effects and repair of tight junctions, ultimately leading to improvements in RE damage.

In this study, we investigated the action targets of HD in relation to RE through pharmacological methods. Our analysis identified MAOA as a critical target for HD in the treatment of RE. We employed Het-1A cells, induced by an acidic culture medium, as an *in vitro* model and treated them with HD. Our results demonstrated that HD restored cell viability, decreased the secretion levels of IL-6, IL-8, TNF-α, and IL-1β, and enhanced the expression of tight junction-related proteins, including occludin, claudin-1, and ZO-1.

Previous research has indicated that Berberine, the primary active component of *Coptis chinensis Franch*, exhibits antidepressant effects linked to the inhibition of MAOA expression [27]. Additionally, MAOA has been reported to be involved in regulating inflammatory processes [28, 29]. In this study, we also found that treatment with HD was associated with a reduction in MAOA expression levels, contributing to the alleviation of damage to Het-1A cells induced by the acidic medium. These results suggest that HD may alleviate the injury of Het-1A cells caused by an acidified medium by inhibiting the expression of MAOA.

Our analysis using HPLC-MS revealed that Stigmasterol is one of the primary active components of HD. However, the current HPLC-MS analysis lacks absolute quantitative results, making it difficult to determine the precise content of Stigmasterol in HD. Therefore, further absolute quantitative analysis is necessary in future research to clarify the exact content of Stigmasterol in HD. This will provide more precise data to support an in-depth exploration of its pharmacological mechanism and clinical application. Stigmasterol is a common plant sterol compound found in soybeans and various traditional Chinese medicines [30, 31]. Research conducted by Wu *et al*. on the anti-anxiety properties of Chuanhu suggested that MAOA has a high affinity for the Stigmasterol phenotype [32]. In line with these findings, we further confirmed the binding of Stigmasterol to MAOA through molecular docking.

Additionally, aligning with the previously reported anti-inflammatory functions of Stigmasterol in osteoarthritis [33], *in vitro* functional studies demonstrated that Stigmasterol mitigated the damage to Het-1A cells induced by acidic culture media and reduced MAOA expression. Notably, a reversal experiment revealed that overexpression of MAOA negated the protective effect of Stigmasterol on cells in the acidic model. These findings suggest that Stigmasterol, the key active component of HD, mitigates damage in Het-1A cells induced by acidic conditions through the inhibition of MAOA expression.

Research has indicated that inhibiting the NF-κB signaling pathway offers protective effects in relation to RE diseases [34-36]. Additionally, the MLCK signaling pathway, which is mediated by the NF-κB pathway, plays a crucial role in maintaining the integrity of the intestinal barrier. Inhibiting this pathway can protect the intestinal epithelial barrier from damage by restoring the tight junctions of the epithelium [25]. In support of this function, our studies showed that Stigmasterol effectively inhibited the activation of the NF-κB-MLCK pathway triggered by an acidic culture medium. However, the overexpression of MAOA counteracted this protective effect. Furthermore, treatment with NF-κB pathway inhibitors (QNZ) reversed the damage caused by the Het-1A acid model, which was exacerbated by MAOA overexpression. These results indicate that Stigmasterol, as an active component of HD, alleviates damage related to RE by targeting MAOA to inhibit the activation of the NF-κB-MLCK pathway.

Given that HD and Stigmasterol effectively inhibited damage to Het-1A cells under the conditions associated with RE, we sought further to explore their potential therapeutic applications for this condition. To assess their effects, we utilized a rat model that is widely recognized for studying the progression of RE [21]. Our experiments confirmed the successful establishment of the RE rat model, as evidenced by macroscopic damage, histopathological changes, inflammation, and barrier dysfunction. Remarkably, both HD and Stigmasterol significantly improved esophageal damage in the RE rats. Furthermore, aligning with our cell studies, we found that these compounds inhibited the activation of the MAOA/NF-κB-MLCK pathway within the esophageal mucosal tissue. These findings underscore the therapeutic potential of HD and Stigmasterol in RE, providing a strong basis for their development as prospective drugs for the clinical treatment of this condition. Previous studies have established that Stigmasterol exhibits significant biological activities, including anti-inflammatory, antioxidant, anti-cancer, and cholesterol-lowering effects. It holds considerable potential in the treatment of diseases such as cancer, metabolic disorders, cardiovascular diseases, neurological diseases, and inflammatory-related diseases [17, 31, 37-39]. However, its bioavailability in the body is relatively low due to its hydrophobic nature [40]. Therefore, future research should focus on optimizing the structure of Stigmasterol and developing efficient delivery systems to explore its potential application and facilitate clinical translation fully.

Additionally, current research indicates that the bioavailability of plant sterols varies depending on the administration form [41]. Subsequently, systematic studies should be conducted to thoroughly explore the absorption, distribution, metabolism, and excretion processes of Stigmasterol under different administration forms. Moreover, a systematic evaluation of the dose-response relationship of Stigmasterol should be carried out. This includes an in-depth exploration of its pharmacokinetic characteristics and clarification of its *in vivo* behavior patterns under various doses, different individuals, and diverse physiological and pathological conditions. Such research will provide a scientific foundation for developing rational clinical medication regimens. 

In summary, this study highlights the multi-component and multi-target effects of HD in treating RE. Notably, Stigmasterol, a recognized plant sterol that reduces the risk of esophageal cancer [16], has been identified as the main active component of HD. Additionally, MAOA has been linked to inflammatory responses [42], and it has been confirmed as one of the core targets of Stigmasterol in RE. Functional studies demonstrate that HD and Stigmasterol effectively alleviated inflammation and repaired tight junction functions in acid-treated Het-1A cells by inhibiting the activation of the MAOA/NF-κB-MLCK pathway, thereby alleviating RE. Further *in vivo* studies support these findings. Our research provides valuable insights for the use of HD in the clinical treatment of RE. However, this study did not perform a systematic evaluation of the dose-response relationship or its pharmacokinetic properties, which are current limitations. Therefore, further testing is required before HD can be widely applied in the clinical treatment of RE.

## CONCLUSION

To sum up, this research showed that Stigmasterol, the main active ingredient in HD, prevented the activation of the NF-κB/MLCK pathway by inhibiting MAOA. This effect led to anti-inflammatory properties and the repair of tight junctions, ultimately resulting in reduced inflammation in acid-treated Het-1A cells. 

## Figures and Tables

**Table 1:** Primers amplified by qRT-PCR.

Name of primer	Sequences (5'- 3')
H-MAOA-F	GCATTTCAGGACTATCTGCTGC
H-MAOA-R	TGGGTTGGTCCCACATAAGC
H-occludin-F	CTCCCTGGCACCGTTGG
H-occludin-R	GCCTGGATGACATGGCTGAT
H-claudin-1-F	ATGACCCCAGTCAATGCCAG
H-claudin-1-R	CAAAGTAGGGCACCTCCCAG
H-ZO-1-F	GAAATACCTGACGGTGCTGC
H-ZO-1-R	GAGGATGGCGTTACCCACAG
H-β-actin-F	ACCCTGAAGTACCCCATCGAG
H-β-actin-R	AGCACAGCCTGGATAGCAAC
R-occludin-F	CCCAGACCACTATGAAACCGACT
R-occludin-R	CAGCCATGTACTCTTCGCTCT
R-claudin-1-F	CAAACTCCGCTTTCTGCACC
R-claudin-1-R	GCTGACGATAGAGCCGATCC
R-ZO-1-F	CCTAATAAGAACAGAGCCGAGCA
R-ZO-1-R	GCAACATCAGCAATCGGTCCA
R-β-actin-F	ACATCCGTAAAGACCTCTATGCC
R-β-actin-R	TACTCCTGCTTGCTGATCCAC

## Data Availability

The manuscript and its supplementary files contain all the data generated or analyzed in this study. The corresponding author can provide raw datasets upon reasonable request.

## LIST OF ABBREVIATIONS

RE: Reflux Oesophagitis
GERD: Gastroesophageal Reflux Disease
PPIs: Proton Pump Inhibitors
TCM: Traditional Chinese Medicine
HD: Huanglian Decoction
NF-κB: Nuclear Factor Kappa B Subunit 1
NLRP3: NLR Family Pyrin Domain-Containing 3
MLCK: Myosin Light Chain Kinase
PPI: Protein–Protein Interaction
HPLC-MS: High-Performance Liquid Chromatography–Tandem Mass Spectrometry
CCK-8: Cell Counting Kit-8
ELISA: Enzyme-Linked Immunosorbent Assay
OD: Optical Density
qRT-PCR: Quantitative Reverse Transcription Polymerase Chain Reaction
TNF-α: Tumor Necrosis Factor-α
IL-1β: Interleukin-1β
IL-6: Interleukin-6
IL-8: Interleukin-8
SDS-PAGE: Sodium Dodecyl Sulfate Polyacrylamide Gel Electrophoresis
TCMSP: Traditional Chinese Medicine Systems Pharmacology Database and Analysis Platform
NCBI: National Center for Biotechnology Information
ESI: Electrospray Ionization
MAOA: Monoamine Oxidase A
